# Failure analysis and recommendations for treatment of posttraumatic non-unions of the distal humerus during childhood

**DOI:** 10.1007/s00068-021-01613-3

**Published:** 2021-02-23

**Authors:** Dirk Walther Sommerfeldt, Peter Paul Schmittenbecher

**Affiliations:** 1 Department of Pediatric Traumatology, Altona Childrens Hospital, Bleickenallee 38, 22763 Hamburg, Germany; 2 Department of Pediatric Surgery, Municipal Hospital, Moltkestr. 90, 76133 Karlsruhe, Germany

**Keywords:** Nonunion, Humerus condylar fracture, Supracondylar fracture, Child

## Abstract

**Purpose:**

Non-unions of the distal humerus are rare complications of common children’s fractures such as radial condyle fractures and supracondylar fractures. The aim of this paper was to update the knowledge about etiology, reasons, management, and results of these troublesome, and sometimes debilitating entities.

**Methods:**

The sparse literature concerning nonunions following condylar or supracondylar fractures was analyzed together with the presentation of some typical clinical cases.

**Results:**

In most of the cases, non-unions were induced by neglect, unstable fixation, too early implant removal, too much revision surgery, and an inconsequent transfer of follow-up algorithms, or combinations of the above. Treatment of non-union should start as early as possible because the effort of required surgery increases with time that the nonunion has been neglected. Often a combination of stable fixation of the pseudarthrosis and correction of the elbow axis are necessary to achieve a satisfying outcome.

**Conclusion:**

In pediatric traumatology, qualified and consequent care for children’s fractures of the distal humerus can prevent rare complications such as non-unions in almost any situation. If such a disturbance of healing is noticed, immediate and adequate, i.e. children specific surgical consequences achieve best results.

## Introduction

In children, fractures usually heal by the high osteogenic activity and the capacity of callus to bridge even bigger defects, distances or unstable situations. Much better blood supply and bone stock result almost always in some kind of fracture consolidation in children. Non-unions in children are mentioned in the current literature [21, 39] with an incidence of 2:1000, and therefore extremely rare, i.e. before the age of 11 years in girls and 13 years in boys. Only if bone quality per se is impaired e.g. by an underlying metabolic bone disease such as osteogenesis imperfecta, osteopetrosis, or the use of bisphosphonates, non-unions can occur more frequently and have to be kept in mind when treating fractures in these patients.

The actual definition of the European Society of Tissue Regeneration in Orthopaedics and Traumatology (ESTROT) describes a non-union as a fracture, which cannot heal without further intervention, independent from previous therapy [6]. Others defined it as a fracture site, at which healing has not taken place for 9 months with no radiologic signs of healing for 3 months [4]. Pace et al. defined condylar non-union in children as lack of callus with fragment migration by 8 weeks after initiation of therapy [25].

Nearly 50% of all non-unions in children appear around the elbow [30]. By the above mentioned, most agreed upon definition true non-unions at the site of the distal humerus during childhood have been described for the epiphyseal part of the humerus and here almost always after fractures of the lateral condyle. Even there, the incidence is extremely low and virtually non-existent at the site of the metaphysis following fractures of the supracondylar region. This fact is strange in so far as supracondylar fractures are the most common fractures of the elbow region during childhood with an incidence of 60–70 per year per 100.000 children [11] and a percentage of all elbow fractures of 58% [12].

In the few cases of true post-traumatic non-unions following a condylar or supracondylar fracture in an otherwise healthy child, we will present that the reasons are almost always a difficult initial injury (open fracture, open reduction), a secondary complication (secondary displacement, wound infection, postoperative instability, revision surgery) or improper rehabilitation protocols (neglection, excessive physiotherapy, too early implant removal). We will report exemplary cases and analyze the possible reasons for failure and the possible therapeutic misconceptions.

## Epiphysis (condylar region): lateral condyle fracture non-unions

### Etiology and fractures/axis at risk

The radial humeral condyle is one of the rare areas developing non-unions in children in the case of inadequate treatment. The incidence is given with 1.4–3% of all radial condyle fractures [25, 29]. A radial humerus condyle fracture is seldom characterized by overtreatment but occasionally by inadequate treatment or neglect [7].

Fractures at risk are

- Complete, but undisplaced fractures without a cartilage hinge if the follow-up controls do not focus consequently on the possibility of secondary displacement (Song type III fractures). The risk of secondary instability is 5.5–18% [9, 14, 27, 28]; and
- Displaced fractures with inconsequent/unstable reduction, fixation and/or immobilisation.

**Fig. 1:**
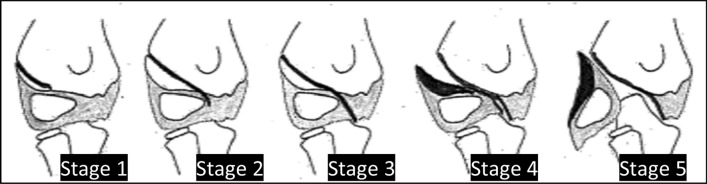
Classification of lateral condyle fractures according to Song [32]

In this context, it is necessary to mention again the characteristics of condyle fractures in the given principal age around 5 years: the ossification center of the capitellum is still small and the cartilaginous part is not visible in the conventional x-ray. The differentiation between Song I (metaphyseal infraction with intact epiphysis), Song II (fracture is running down to the capitellum but with an intact cartilage hinge) and Song III (complete, but undisplaced fracture) (Fig. 1) is impossible without MRI, ultrasound [36] or arthrography [9, 32, 38]. Therefore, if in young children the initial x-ray shows the well-known very fine metaphyseal fracture line parallel to the cranial edge of the capitellum, nobody can decide with sufficient certainty whether a chondral hinge is preserved or not. A conventional x-ray control at day 5–7 without cast is recommended in all these cases [8, 14] and seems to be the most practical of all radiological options. In case of any secondary displacement, the fracture is unstable und should be fixed to prevent further displacement. If this is not consequently done healing may be delayed or non-union develops because of non-respected instability. Displacement later than day 5–7 is very unusual and 2–3 further weekly controls as recommended by Abzug et al. [1] are dispensable!

If in older children the initial x-ray shows without doubt a definitely complete, but undisplaced fracture, the control is necessary in the same way to realize or exclude secondary displacement. The most important point is to know about these dynamics! To prevent this complication in complete fractures, some authors recommend percutaneous fixation of complete, but undisplaced fractures [1] what means a prophylactic osteosynthesis. It is questionable whether this approach is indicated in all undisplaced fractures to take care of an inadequate control algorithm [19].

**Fig. 2:**
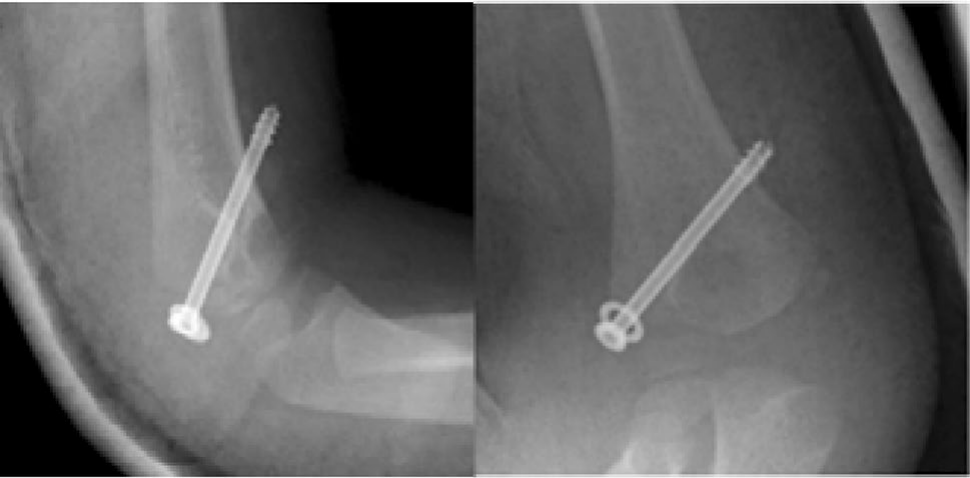
Condyle fracture fixed with a too long screw. The whole thread is outside the bone, fixation is unstable

**Fig. 3:**
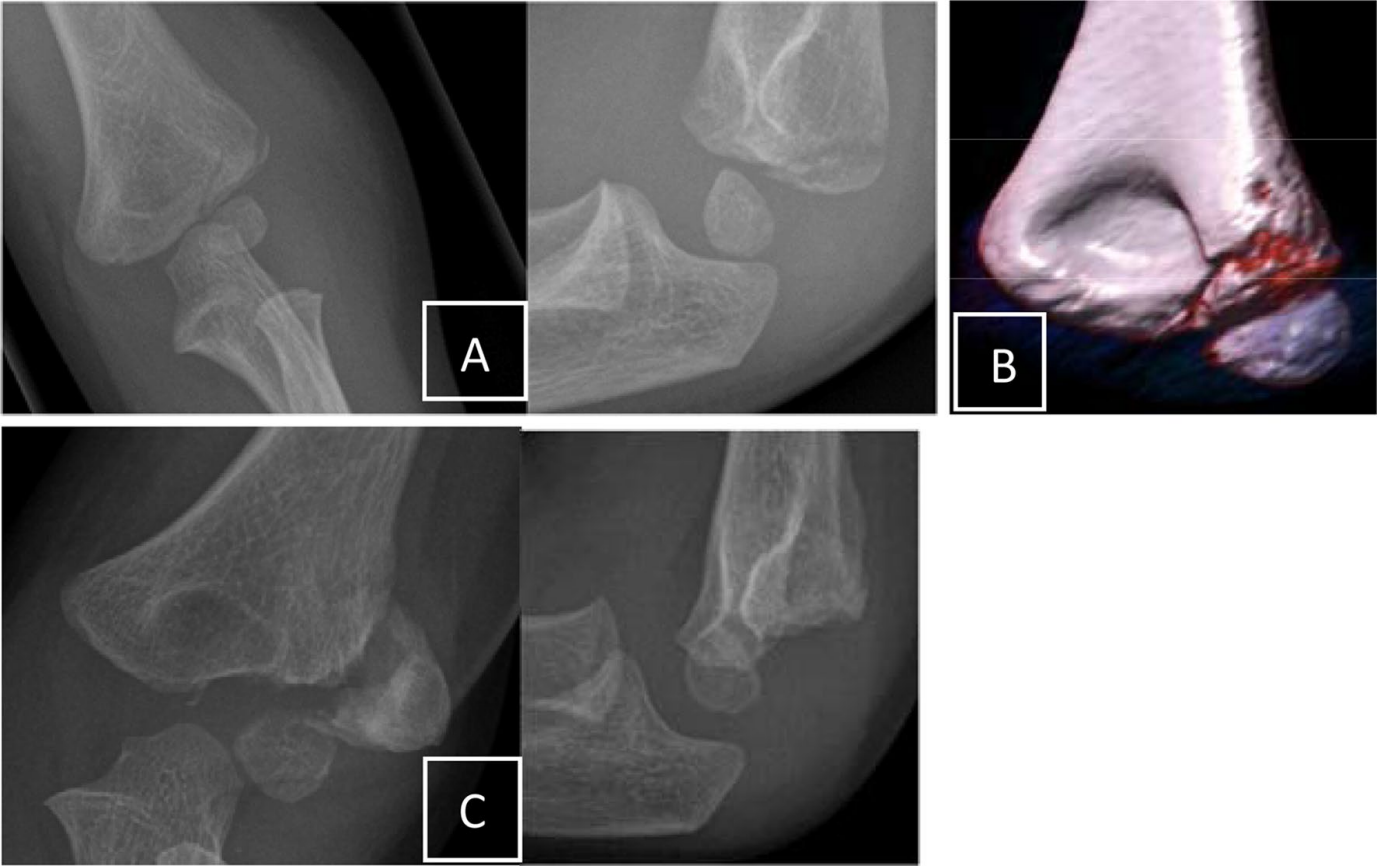
**a** Typical undisplaced condyle fracture in a small child. **b** The fracture characteristics were additionally, but unnecessarily documented with CT. No control at day 5–7 but 4 weeks later with the secondarily displaced fragment. Waiting for whatever. **c** Definite non-union 1 year later

If an osteosynthesis is realized, inadequate additional immobilisation in the use of K-wires, an unstable fixation with K-wires or screws (Fig. 2) or too early removal of the implants (Fig. 3) can initiate a similar process of secondary displacement with the risk of delayed non-union [24].

### Symptoms and diagnostics

Clinical signs are loss of motion, lateral elbow spurring or bump, valgus deviation of the elbow axis and pain by the given instability. Range of motion may be compensated by some mobility within the non-union. The cosmetic change is remarkable. Over time ulnar nerve palsy may develop [14, 34, 35].

Clinical examination (ROM, elbow axis, stability, ulnar nerve irritation) and a conventional x-ray are sufficient in most cases. The fragment usually slides lateral and secondarily proximal and is often in an oblique position. Fishtail deformity may be present. CT or MRI scans usually cannot give any important additional information. The question of fragment mobility cannot be answered clinically and has to be proven intraoperatively. Perhaps the visualisation of the articular surface and the documentation of the chondral structures may be interesting making MRI an option.

### Timepoint of intervention

Historical case reports found unlimited daily activity even without revision of a condylar non-union [22]. With good function and without pain the need fot=r for operative intervention can be discussed. But today few patients and/or parents will accept the risk of relevant limitations in the further course of such a situation if it is diagnosed in children.

Time of correction should be as soon as possible following the diagnosis to prevent further displacement. If non-union is found, waiting can lead to further displacement, pathological remodeling of adjacent bones and articular surfaces, and therefore higher surgical expense only.

### Methods

**Fig. 4:**
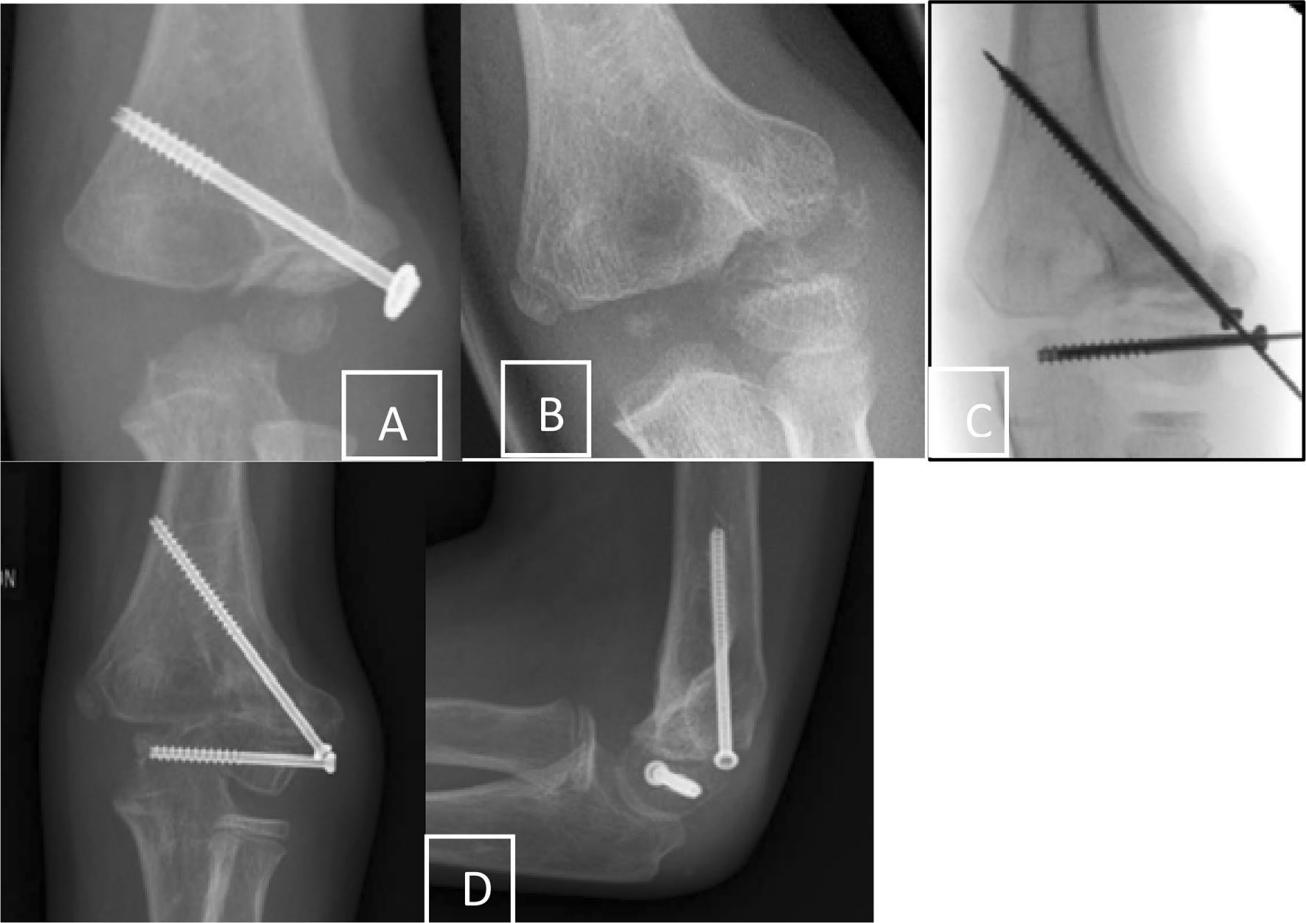
**a** Condyle fracture with screw osteosynthesis. Removal without signs of healing. **b** Some times later with a petty injury pseudarthrosis was detected and **c** fixed in situ with two screws. **d** Finally full functional recovering with radial spur

Within the first 4 months following the initial trauma and as long as the fragment did not slip in an oblique position percutaneous screw fixation is an option with good success [15] (Fig. 4). The authors were successful with this kind of procedure in children with a median time of 16 weeks from initial presentation, but unsuccessful with cases that presented themselves at an even later timepoint.

In cases with more displacement or proximalisation of the fragment, it is not recommended to extensively mobilize the fragment to achieve a complete reduction [33]. On one side the articular surface cannot be restored perfectly, or if there is some chondral regeneration, intraarticular osteotomy and reduction induces a new incongruity of the articular surface. On the other side extensive mobilization of the condyle includes the risk of avascular necrosis [1]. Therefore, the most recommended method is the fixation in situ with only limited open debridement of the non-union and stabilization with two screws [15, 26]. Park et al. treated 16 consecutive patients with in situ fixation consisting of minimal curettage and screw compression within 3–12 months since the initial fracture. The growth plate should be respected if possible even if the local growth potential is low and the whole manipulation may induce ossification and closure of the physis (Figs. 3, 4).

**Fig. 5:**
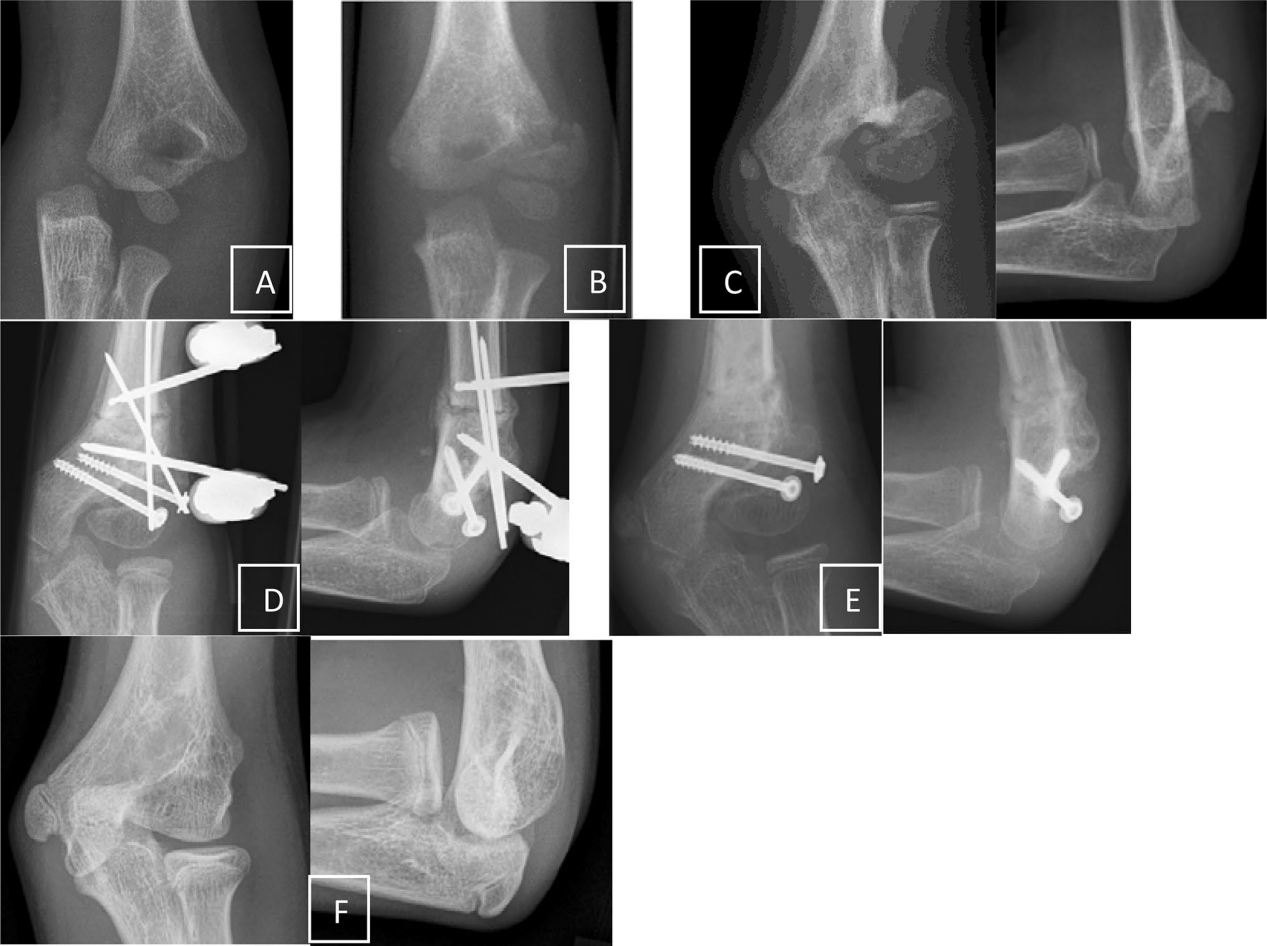
**a** Severe traumatic condyle displacement. **b** Closed reduction without osteosynthesis. **c** Long observation with definite non-union and relevant valgus deformity. **d** Screw fixation following careful debridement and mobilization and additional supracondylar osteotomy with Slongo’s fixator technique during the same surgery. **e** Stepwise hardware removal. **f** Longtime result in the age of 13 years with free ROM and 8° valgisation compared to the other side without request for further correction

If the elbow axis has additionally changed over time to a relevant amount and valgus deformity is more than 20° compared to the healthy side, an additive supracondylar osteotomy is necessary. Most authors prefer supracondylar dome osteotomy to prevent a translational effect [16, 33]. Tien et al. achieved uneventful healing of all non-unions as well as all dome osteotomies. The functional results were excellent or good in 6/8 patients, classified according to a score described by Dhillon et al. [5] including ROM, Humerus-Ulna angle, medial shift and pain or weakness. Alternatively, a closed wedge osteotomy corrects the axis and may be fixed with K-wires, screws or preferentially with an external fixator in Slongo’s technique to make immediate movement possible without any immobilization [31] (Fig. 5). Using the external fixator technique some translation of the distal fragment prevents the overlapping of the fragments as well.

If ulnar nerve irritation is the main symptom, anterior transposition of the ulnar nerve may be indicated [18] independent from correction of the axis. Preferentially the non-union should be diagnosed and fixed before ulnar nerve irritation takes place.

Methods such as extensive distalization of the pseudarthrotic fragment with interposition of a corticospongious bone block [17, 18], with a free vascularized iliac crest bone craft [3] or with vascularized humeral periosteal flap [2] seems to be seldom necessary.

### Results and problems

The quality of the results depends on the age of the patients, the amount of preoperative symptoms and the number of postoperative complications [34, 35]. If the range of motion was limited before, mobility will be better in most of the cases, but moderate limits will often remind. If the range of motion was based on mobility within the non-union in a relevant amount, postoperative temporary restriction has to be feared.

### Summary

Condylar non-unions can and has to be prevented by consequent management of condylar fractures according to the algorithm given in the guideline [8]. The earlier delayed healing is realized the smaller is the amount of necessary surgical manipulation. Fixation in situ is the recommended management with additive supracondylar osteotomy if need be. Ulnar nerve transposition is dispensable in the early detection of delayed healing. Longtime sequelae are possible. Best prevention is to avoid neglection of the initial instability by regular controls.

## Metaphysis (supracondylar region): supracondylar fracture non-unions

### Etiology and fractures/axis at risk

There are several factors contributing to the development of a non-union following a supracondylar fracture in children, starting usually with the description of a “difficult” fracture:

- Disruption of blood supply in critical areas;
- The subjectively felt need for an open reduction leading to
- Missing fracture hematoma leading to
- Low concentration of mesenchymal pluripotent stem cells and growth factors;
- Concomitant vascular and/or nerve injury;
- Complex regional pain syndrome (CRPS);
- Secondary instability due to inadequate implant placement (suboptimal osteosynthesis) or
- Too early removal of implants, i.e. before radiologically completed fracture healing, and
- Early revision surgery leading to more complications.

In our cases, especially the first and last bullet point (vascularity and early implant removal) seem of importance and warrant further attention.

#### Vascularity

In general, the distal humerus has a rich anastomotic blood supply through epiphyseal and metaphyseal vessels entering the bone from all sides [13]. However, a so-called watershed area has been described for the supracondylar metaphyseal region, where the humerus is thin in diameter and is reached by the above-mentioned vessels only as a “last meadow” or postcapillary fashion [37]. If this particular blood supply is severed by the injury first, by an open reduction second, and by revision surgery third, even the abundant collateral vessels around the pediatric elbow may not be able to sustain proper oxygenation and metabolism for fracture healing in a timely fashion.

#### Implant removal

**Fig. 6:**
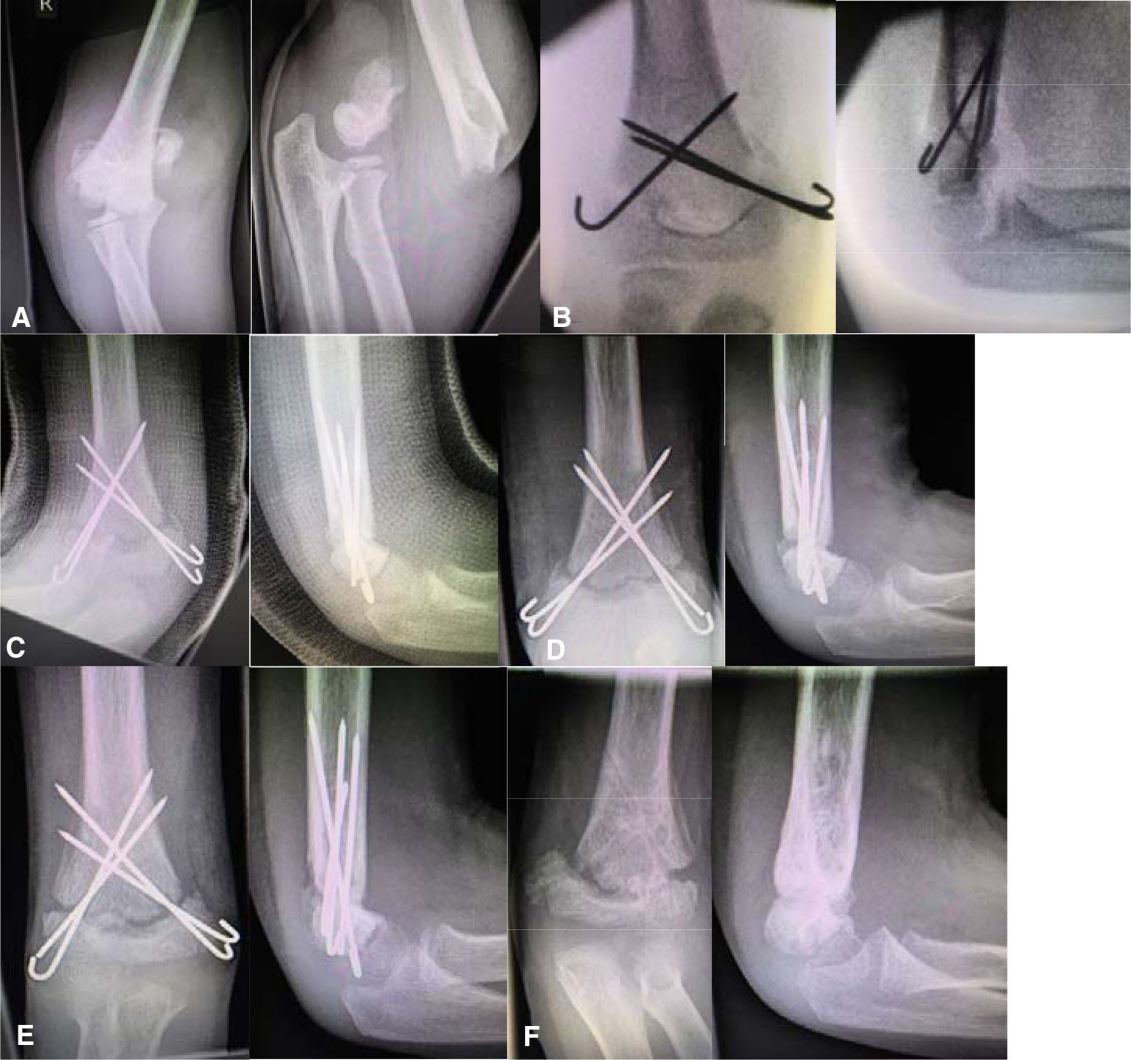
Development/fabrication of non-union. In chronologic order different steps of treatment/decision-making that lead to non-union can be retraced. **a** Post injury. **b** Post closed reduction and stabilization. **c** Post open revision surgery due to insufficient reduction in the sagittal plane. **d** Four weeks control. **e** Eight weeks control. **f** After implant removal despite insufficient callus formation

Many textbooks give exact schedules and timeframes, as to at which timepoint post-fracture cast removal, physiotherapy, return to sports, and implant removal should take place. What is sometimes not clearly mentioned is the fact that these intervals are mere suggestions and very often have to be adjusted to the healing process of the individual patient. Therefore, and as you will see later on if you cling to the suggested timeframes in a too rigid fashion the consequences can be detrimental to the patient’s recovery. This holds especially true for the timepoint at which implant removal should be considered. One would think that it is obvious to not remove implant materials (if there is no infection or other soft-tissue problem) before fracture healing has taken place and can be validated by clinical examination and radiological diagnostics. However this simple rule was not always followed and hardware removal was scheduled before fracture healing had occurred (Figs. 6, 8).

Depending on the quality of the primary reduction and on the duration of the non-union deformity can occur in any of the three axes in space separately or in combination. The position of the distal fragment in a non-union is dependent on the quality of the pseudarthrosis (stiff or loose, hypertrophic or atrophic) and on the pull of the attaching muscles. Most commonly, the distal fragment in a non-union “behaves” similar to the one after injury in the frontal plane, so a varus deformity is more common than a valgus deformity, with the valgus deformity being more prone to clinical symptoms such as ulnar nerve palsy. In the sagittal plane, however, flexion deformity due to brachioradialis and brachialis muscle pull is more likely than extension deformity. Similar to a supracondylar fracture you may encounter varying degrees of rotational deformity, with the distal fragment more likely being externally (posterolateral) rotated than internally (posteromedial). In addition to the described redislocating forces asymmetric growth and hypertrophic callus formation can also influence the shape and degree of the deformity, especially in cases, where the non-union has been established for many months or even years. In these patients, over the course of time you may see a varus deformity due to stimulated bone formation laterally in the beginning turning into a valgus deformity later on when the non-union becomes atrophic and partial osteonecrosis occurs at the lateral condyle.

### Symptoms and diagnostics

Patients presenting with a long-standing non-union can be surprisingly pain-free and compensating their loss of function in the elbow joint by mobility within the pseudarthrosis (“false joint”). As mentioned above, ulnar nerve tension in a valgus deformity can lead to pain or senso-motory deficits. The main symptom, however, remains the cosmetic appearance of the elbow together with the loss of function of the elbow joint. Depending on the deformity there is usually a flexion and/or extension deficit to a varying degree.

### Timepoint of intervention

It was said and written for a long time and until recently that the best time point for correction of posttraumatic deformities during childhood in general was after the closure of the adjacent growth plates. For this reason, additional deformities having developed within a long-standing non-union were often neglected until adulthood [23]. However, this does not hold true anymore and we recommend a more differentiated approach to these problems. Since we seem to have a fairly good understanding now as to which deformities in terms of localization and degree will be able to remodel due to remaining growth we can also predict which deformities will not correct through appositional and enchondral growth processes. In addition, we have found out that in the case of a deformity in one axis that will not be able to correct itself through remodeling the remaining growth can lead to much more complex and demanding situations for the patient and the surgeon.

It is for these reasons that we recommend to perform correction of additional axial deformity in children if at all possible within the same session that we treat the non-union, especially if the existing posttraumatic deformity has limited or no potential for spontaneous remodeling to a functionally satisfying outcome.

This holds especially true for the distal humerus where bone growth is limited per se and remodelling of a posttraumatic deformity following a supracondylar humerus fracture can only be expected if the deformity is in the sagittal plane, which is the axis of movement of the elbow joint. In the literature and by personal observation we can see correction of flexion and extension deformities up to 30° and until the age of 8 years. If the deformity is greater, the axis of deformity different from the sagittal plane or a complex multidimensional one as is always the case in longstanding deformities, and if the patient is older than 8 years we recommend deformity correction as soon as possible nowadays to prevent the progress of the deformity into a more complex, i.e. more-dimensional one.

Taking these experiences into account it is safe to say that for delayed and non-unions an early timepoint for revision is mandatory and that the accompanying deformity, be it a cubitus varus or valgus, a hyperextension or -flexion, a rotational deformity, or, as in most cases, a combination of these, should be corrected surgically in one session, if technically possible. The patient may be doing surprisingly well with the non-union in some instances and it can be difficult to persuade the parents to go forward with surgery if their child is pain-free and moving its arm well, but a persisting non-union at the supracondylar level is a debilitating deformity and will not serve the patient well in the future in terms of future physical work and recreational activities.

### Methods

For delayed unions, i.e. insufficient radiologic consolidation after 3 months, in which the primary crossed K-wire osteosynthesis seems to be the problem in terms of stability, the lateral external fixator [31] is an excellent option to increase stability, even if bone stock—quite possibly due to multiple drill holes in the two fragments—seems to be poor.

**Fig. 7:**
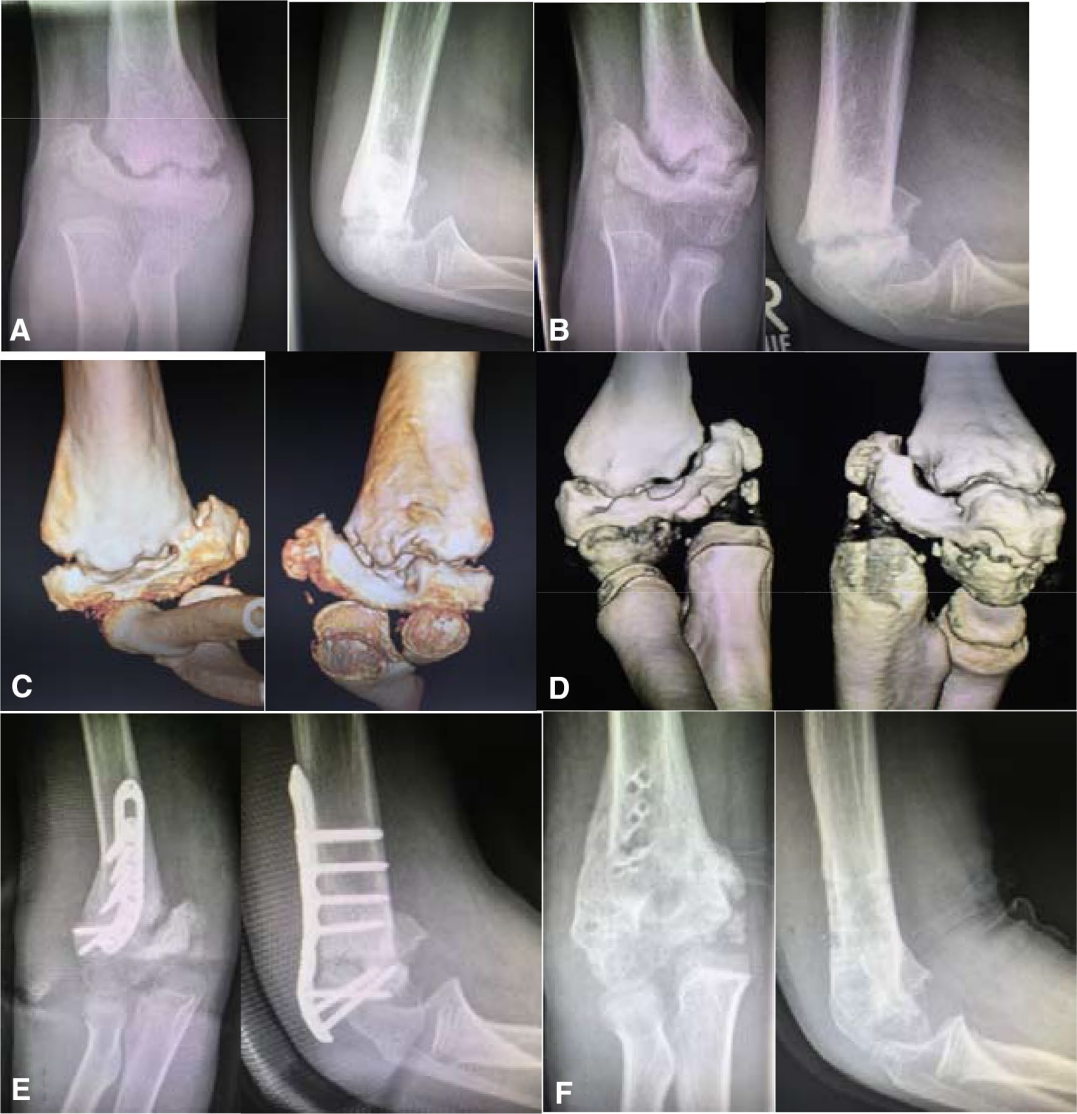
**a** Images after 6 and **b** after 12 months. **c** 3D-CT after 12 and **d** after 18 months. **e** Postoperative control after radial distal humerus plating via triceps-sparing approach and cancellous autograft. **f** After implant removal 1 year after plating and bone-grafting

**Fig. 8:**
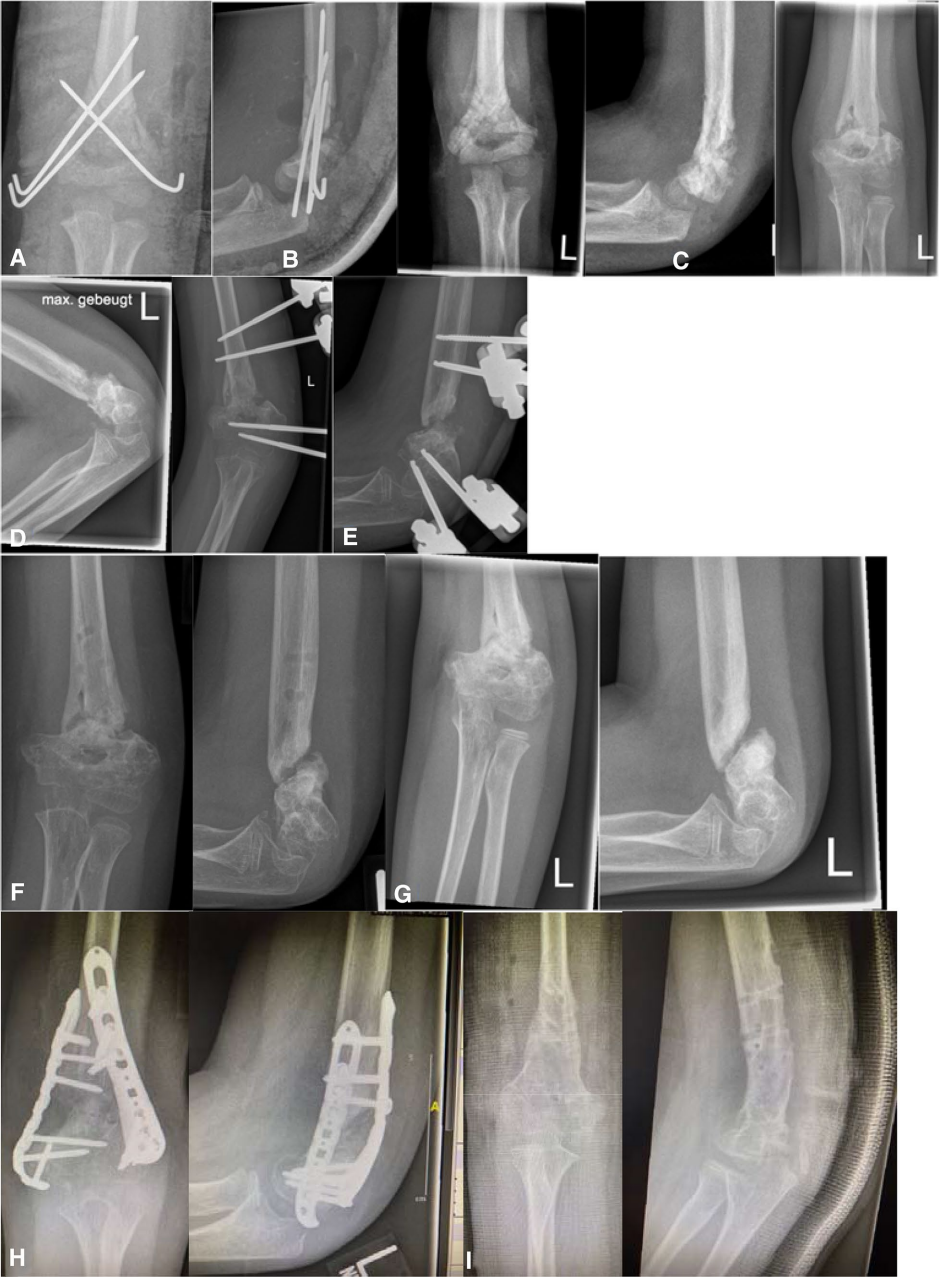
Two times (!) premature implant removal leading to long-standing non-union with gross limitation of joint movement and additional movement within the false joint (non-union) and entrapment of the ulnar nerve. **a** Post injury lateral. **b** Postop open (!) reduction and crossed K-wires. **c** First implant removal before consolidation, reason unknown. **d** Non-union after 6 months. **e** Decision for external fixator as salvage procedure No. 1. **f** Second implant removal before consolidation, reason unknown. **g** Ebonized non-union 2 years post trauma. **h** Salvage procedure No. 2, double-plating 2.7/3.5 mm plus autogenous bone graft + 3D deformity correction. **i** Post plate removal after non-union consolidation 1 year postop with still grossly limited range of motion but union of the distal humerus after a total of 3 years post trauma

If there is a true non-union with no signs of consolidation progress over the course of 3 months, and as in Fig. 8 presented, the external fixator has already been applied as a salvage procedure, there is usually an accompanying major deformity which warrants open osteotomies or resection of the pseudarthrotic tissue. In addition, and especially if there is an atrophic situation, we strongly recommend autogenous bone grafting (iliac crest, proximal ulna) in addition to a plate osteosynthesis. In the era of anatomically preformed plate systems the smaller and low-profile designs are very well suitable for children even as young as 6 years (Figs. 7, 8).

### Results and problems

If the pseudarthrotic deformity is corrected at an early timepoint and the corrective surgery is the first revision procedure, results will almost be satisfying and successful if no additional mistakes are made postoperatively in terms of rehabilitation or implant removal (Fig. 7). If the accompanying deformity has reached a three-dimensional degree over the course of many months or years it may require more than one intervention to achieve consolidation of the distal humerus and free range of motion may not be achievable in these cases.

### Summary

True non-unions of the supracondylar region of the humerus are ten times rarer in children than in adults. When caught early, i.e. still as delayed unions within 6 months post-injury results after revision surgery are usually good with high consolidation rates over the course of 3–6 months and good to excellent functional results. Closed reduction and use of a monolateral external fixator can usually get the job done with no need for open debridement and/or autogenous bone transplants. Non-unions are much more difficult to treat. An open correction of the accompanying deformity together with resection of the pseudarthrotic tissue and bone grafting is necessary in our experience. Distal low-profile 2.4/2.7 mm preformed plates are safe and reliable implants to achieve a union over a timecourse up to one-year post revision surgery.

If these considerations are taken into account delayed unions can heal with no loss of range of movement (ROM) within the elbow joint. Non-unions may consolidate but may render elbow function less than normal with flexion and/or extension deficits to a varying degree. It is important to be open and frank about the duration and invasiveness of the surgery and the achievable results in terms of function to the patient and the parents before doing the preoperative planning.

### Final conclusions for both entities

Delayed and non-unions are rare challenges in the treatment of fractures in children and even more so following condylar and supracondylar fractures of the humerus. It is important to mention that in our experience all non-unions developed over a long time period while being either neglected or mistreated during that time. In other words, we could not identify a single case where pseudarthrosis developed spontaneously after correct surgical or conservative treatment protocols according to existing guidelines.

By adhering to the principles described by Lexer [20] and Ilisarov [10] who are derived from the experiences in adults one can be successful in treating these entities in children successfully just as well or even better due to better healing potential of the bone and better vascularity at the site of former injury. Stability seems to be the key, which can be usually achieved by closed revision surgery and a change towards a more stable implant.

In true non-unions vascularity and stability are the main factors following resection of the pseudarthrosis in a hypertrophic situation or curettage and bone grafting in an atrophic situation again, stability has to be provided, and small low-profile plating systems have been a good choice in the cases we have treated so far.

It is of importance to discuss the length and invasivity of the proposed treatment with the patient and the parents and to mention the fact that function of the elbow joint may not be fully restorable and some deficit may persist permanently.
